# Iron-based single-atom electrocatalysts: synthetic strategies and applications

**DOI:** 10.1039/d0ra08223f

**Published:** 2021-01-14

**Authors:** Qinglei Liu, Yongfei Wang, Zhizhi Hu, Zhiqiang Zhang

**Affiliations:** Key Laboratory for Functional Material School of Chemical Engineering, University of Science and Technology Liaoning 185 Qianshan Zhong Road Anshan 114044 P. R. China zzq@ustl.edu.cn wyf8307@ustl.edu.cn; School of Materials and Metallurgy, University of Science and Technology Liaoning 185 Qianshan Zhong Road Anshan 114044 P. R. China wyf8307@ustl.edu.cn

## Abstract

The performance and cost of electrocatalysts play an important role in the development and application prospects of energy conversion technology. Single-atom catalysts (SACs) have constituted a new frontier in the field of catalytic science in recent years. As a non-precious metal, iron (Fe)-SACs show great potential in the field of electrocatalysis, which is comparable to or even better than the performance of precious metal catalysts. However, a robust, generic synthetic strategy toward atomically dispersed Fe catalysts is still lacking, which is still a formidable challenge to maintain the dispersion of Fe atoms at high temperatures and to obtain high catalytic activity. In this review, the latest progress in the synthesis of Fe-SACs is introduced and summarized, and the electrochemical applications of Fe-SACs are further summarized and discussed. Herein, the relationship between the structural characteristics and performance of Fe-SACs is further introduced and discussed. Finally, the existing problems and development prospects of Fe-SACs are discussed.

## Abstract 2

The performance and cost of electrocatalysts play an important role in the development and application prospects of energy conversion technology.

## Graphical abstract

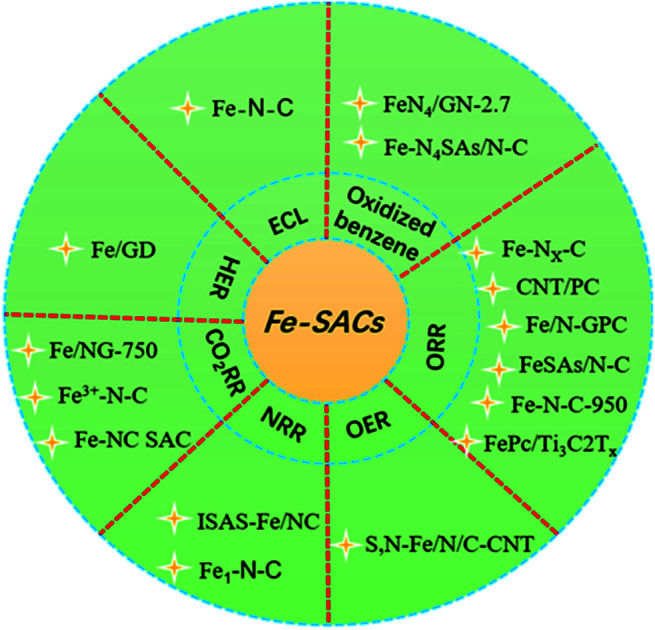

## 1. Introduction

**Fig. 1:**
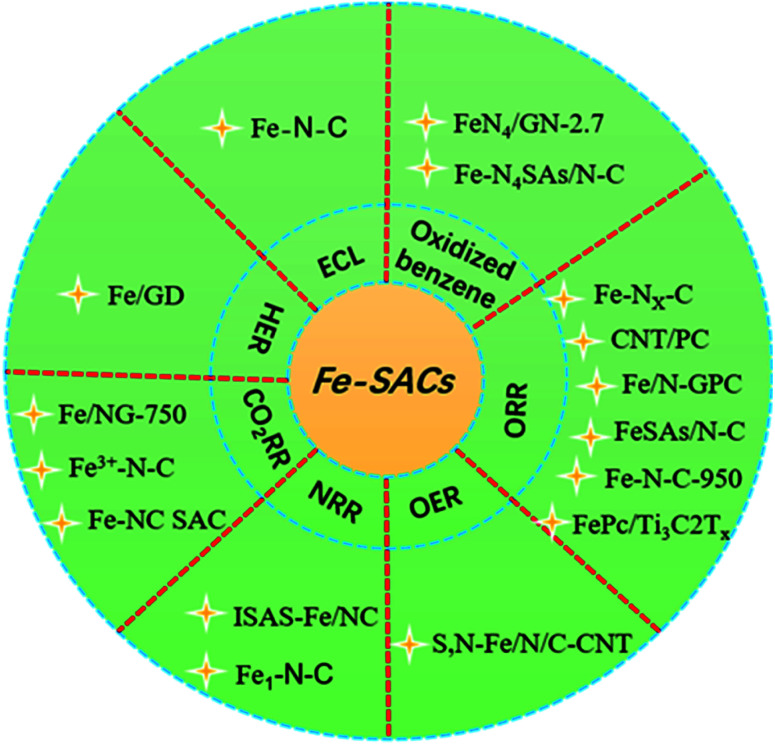
Applications of Fe-SACs.

Single-atom catalysts (SACs) combine the advantages of heterogeneous catalysts and homogeneous catalysts, and bridge the gap that exists between them with their unique performance. It is expected to become a bridge between homogeneous catalysis and heterogeneous catalysis, which has been a rising star in the field of catalysis in recent years.^1,2^ SACs have the advantages of maximum atomic utilization, uniform active sites, adjustable electronic environment, high catalytic activity and selectivity, better stability, and excellent recyclability. However, whether in the process of preparation or in the subsequent application process, the strong trend of migration and aggregation of active atoms and the controllable preparation of SAC is still a challenging task.^3–11^ Transition noble metal monatomic catalysts such as ruthenium (Ru), silver (Ag), rhodium (Rh), palladium (Pd), iridium (Ir), platinum (Pt), and gold (Au) have been widely used in various electrochemical reactions such as oxygen reduction reactions (ORRs), hydrogen evolution reactions (HERs), carbon dioxide reduction reactions (CO_2_RRs), and oxygen evolution reactions (OERs).^12–15^ However, the high cost and low natural abundance of precious metals seriously hinder the wide range of applications of these technologies. Therefore, the monatomic catalyst of non-precious metals has become the research focus of the majority of scholars. The latest research shows that iron (Fe)-SACs can be used in a variety of electrocatalytic reactions such as HERs, ORRs, nitrogen reduction reactions (NRRs), and CO_2_RRs (Fig. 1), exhibiting the same or even better catalytic performance than precious metal catalysts (the specific performance of the catalyst is described in detail in the section describing applications of Fe-SACs).^8,16–19^ These results provide a new possibility for the substitution of precious metal catalysts and show a great application prospect.

To date, the preparation of Fe-SAC and its application in the field of electrocatalysis have not been reviewed. Therefore, it is necessary to comprehensively review the determinants of its preparation method, structure, and activity to provide a basis for the design of this type of catalysts and promote further research and development in this field. In this article, we present an in-depth understanding of Fe-based SACs from the following aspects: (1) the relationship between structural characteristics and performance, (2) the application in the field of electrocatalysis, and (3) the preparation strategy of Fe-based SACs. Finally, we look forward to the prospects and challenges of Fe-SACs.

## 2. Active sites of monoatomic iron catalysts

Several factors affect the activity of Fe monatomic catalysts; however, it is still a great challenge to clarify their structure–property relationship. According to the current research progress of Fe monoatomic catalysts, the active sites of Fe monatomic catalysts are closely related to the local environment of Fe atoms, that is, the coordination number and electronegativity of adjacent atoms. Therefore, most of the research studies are mainly focused on Fe–N–C catalysts. Although the Fe–N–C catalyst shows excellent catalytic performance, it usually leads to the formation of Fe active sites with different coordination configurations. The heterogeneity of these structures and components makes the determination of the active sites and the relationship between the structure and catalytic performance very complicated. Fe atoms have a large number of empty d orbitals and different types of coordination structures, thus according to the number of Fe atoms and coordinated nitrogen atoms in the active sites, there are mainly four types of active sites of Fe monoatomic catalysts: Fe–N_4_, Fe–N_2_, Fe–N_6_, and Fe–N_*x*_, among which Fe–N_4_ is the most reported active site. The structure of the carrier comprising the location of these active sites and the surface loading rate also has a significant impact on the catalytic performance. Different types of Fe active sites show different catalytic properties for different electrocatalytic applications. In recent years, research efforts have been devoted to the theoretical simulation and experimental verification of Fe-SACs, mainly to establish the relationship between the local atomic structure and catalytic performance, in order to explore its potential application in electrocatalysis.

**Fig. 2:**
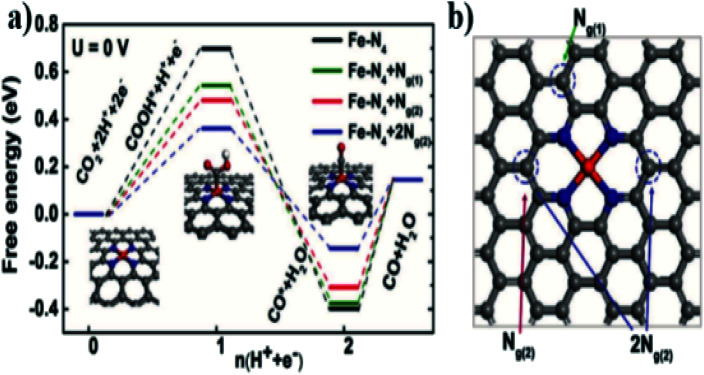
Theoretical calculations and proposed mechanism on the nitrogen-coordinated Fe catalytic site. (a) Free energy diagram of electrochemical CO_2_ reduction to CO on Fe–N_4_ moieties embedded on graphene sheets. (b) Top view of the optimized structures of Fe–N_4_ moieties embedded on graphene layers and potential nitrogen-substitution.

The monatomic Fe catalyst dispersed on nitrogen-doped graphene (Fe/NG) reported by Zhang *et al.*^20^ consists of a pyridine-type Fe–N_4_ structure, and nitrogen doping plays an important role in improving the conversion of CO. A small amount of Fe atoms loaded in NG could form a Fe–N_4_ site capable of enhancing the adsorption of CO_2_ and improving the activity of CO_2_. Fig. 2a and b demonstrate that the mechanism includes the following steps: (1) CO_2_ + * + H^+^ + e^−^ → COOH*, (2) COOH* + H^+^ + e^−^ → CO* + H_2_O, and (3) CO* → CO + *, where * represents the active site on the surface of the catalyst. Moreover, the three free energy diagrams with different nitrogen doping configurations of graphite indicate that the nitrogen substitution on graphene improves the catalytic activity of the Fe–N_4_ moieties by lowering the energy barrier of COOH* formation, as well as facilitating the CO* desorption step.

**Fig. 3:**
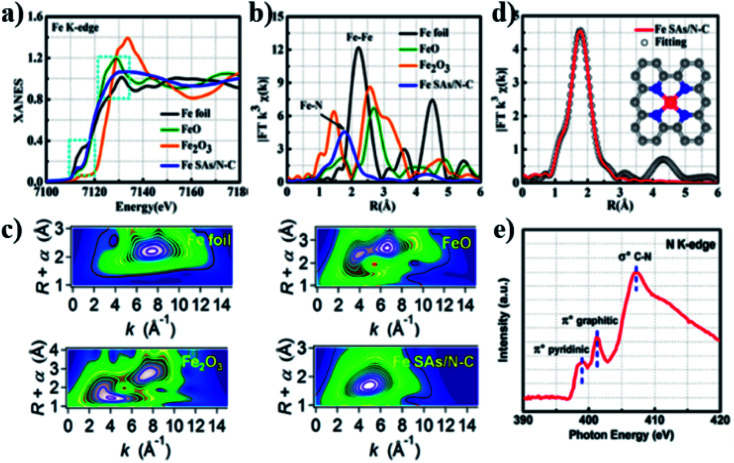
(a) XANES and (b) FT-EXAFS curves of Fe SAs/N–C and references at the Fe K-edge. (c) WT-EXAFS of the Fe foil, FeO, Fe_2_O_3_ and Fe SAs/N–C. (d) FT-EXAFS fitting curve of the Fe SAs/N–C sample. Inset: atomic structure model of Fe SAs/N–C, Fe (red), N (blue), and C (gray). (e) N K-edge NEXAFS spectrum of Fe SAs/N–C.

The Fe monoatomic catalyst Fe SAs/N–C reported by Yang *et al.*^21^ consists of a single Fe–N_4_ as the active site and shows outstanding performance toward ORRs. They used extended X-ray absorption structure (EXAFS) and X-ray absorption near-edge structure (XANES) to study the coordination environment and chemical state of Fe–SAs/N–C. Fig. 3a shows that the single atom of Fe in Fe SAs/N–C may be in a low-valence state (0 < *δ* < 2). Fig. 3b demonstrates that the main peak of the catalyst corresponds to Fe–N coordination, and its central position is about 1.5 Å. The results indicated that Fe atoms were loaded onto N-doped carbon nanosheets in the form of single atoms. Fig. 3c illustrates that the wavelet transform (WT) diagram of Fe SAs/N–C shows only one intensity maximum at 5 Å^−1^, which can be specified as Fe–N coordination. The EXAFS measurement and fitting curve (Fig. 3d and Table 1) indicate that the Fe single atom has a good Fe–N_4_ structure. Three peaks of pyridine N π* (399.5 eV), graphitized N π* (402.4 eV) transition, and the formation of C–N σ* bond (408.5 eV) were observed in the N-K edge spectrum shown in Fig. 3e.

**Table 1:** Structural parameters extracted from the Fe K-edge EXAFS fitting (S02 = 0.74)

Sample	Scattering pair	CN	*R* (Å)	*σ*_2_ (10^−3^ Å_2_)	Δ*E*_0_ (eV)	*R* factor
Fe SAs/N–C	Fe–N	4.2	2.03	8.9	4.5	0.0078
Fe foil	Fe–Fe1	8*	2.48	5.9	0.5	0.0057
Fe foil	Fe–Fe2	6*	2.84	7.2	0.5	0.0057

**Fig. 4:**
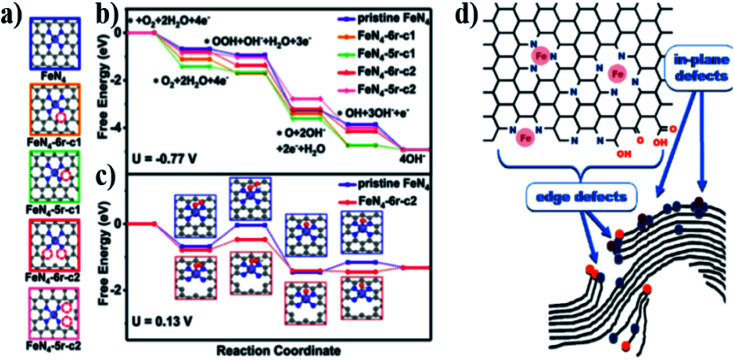
(a) Five possible atomic configurations with different cracking degrees (6r or 5r refers to the six- or five-membered Fe–N heterocyclic ring, respectively; c1 or c2 refers to the cleavage of one or two C–N bonds adjacent to Fe–N_4_, respectively). Free energy diagram (b) at *U* = −0.77 V and (c) at *U* = 0.13 V (*vs.* SHE). (d) Schematic of the transition metal–containing nitrogen defects in graphene sheets (Fe–N_*x*_) and other possible defects that may have catalytic activity in ORRs and its component reactions.

The activity of the ORR was enhanced by selectively breaking the C–N bond at the edge of the active site of Fe–N_4_.^22^ Five Fe–N_4_/C models with different edge N atoms were proposed through density functional theory (DFT) (Fig. 4a). At the equilibrium potential *U* = −0.77 V (*vs.* SHE) (Fig. 4b), the free energy change (Δ*G*) of the four-electron reduction path was negative, thus it was exothermic. However, Fig. 4c shows that the first electron transfer sub-step (*O_2_ + H_2_O + e^−^ → *OOH + OH^−^) has a maximum positive Δ*G*; therefore, this step is a step that determines the rate of the reaction process. FeN_4_-6r-c1 and FeN_4_-6r-c2 have lower values of Δ*G*, which are 0.31 and 0.32 eV, respectively. However, for step 5 (OH* + e^−^ → O^−^), FeN_4_-6r-c1 showed higher resistance (0.72 eV) and better overcoming ability for FeN_4_-6r-c2 with a higher cleavage degree (0.13 eV). In general, the defect FeN_4_-6r-c2 is located at the edge of the cavity and has the lowest total reaction free energy change (0.32 eV). These results confirm that the C–N structure changes the charge density distribution and electronic structure of the active site.

Serov *et al.*^23^ believed that the active site of the catalyst not only comprised Fe–N, but also included some in-plane and edge defects. Fig. 4d shows the schematic of in-plane Fe–N_*x*_ defects and edge defects in a hypothetical graphene sheet. Edge defects include nitrogen and oxygen, and they may also contain Fe–N_*x*_. All these defects may act as potential active sites in ORRs.

**Fig. 5:**
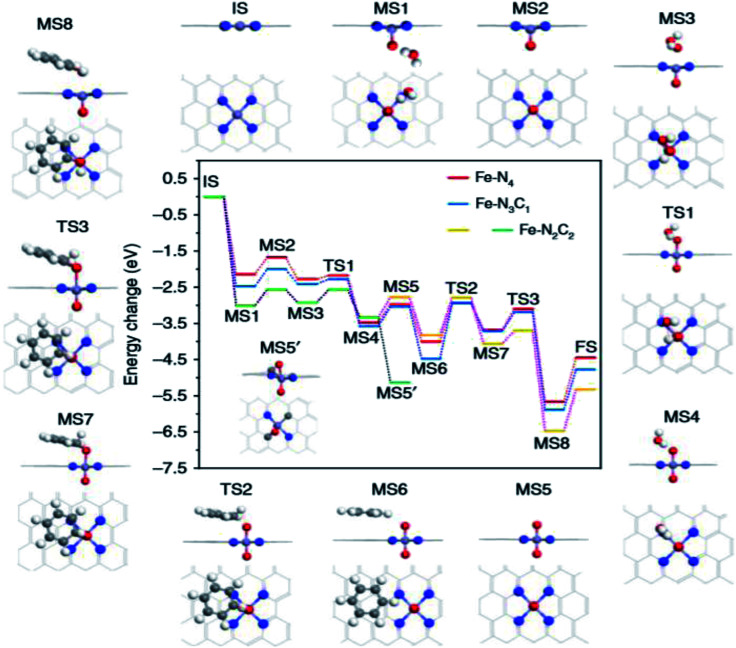
DFT calculation. Energy diagram of benzene oxidation on Fe–N_*x*_C_*y*_ SAs/N–C catalysts with the related reaction configuration on Fe–N_4_ surrounded (IS: initial catalyst, MS1: the first H_2_O_2_ cleavage adsorption, MS2: Fe=O configurations, MS3: the second H_2_O_2_ adsorption on the opposite side, TS1: transition state of the formation for the second H_2_O, MS4: the second H_2_O adsorbed configuration, MS5: O=Fe=O moiety, MS5′: the abnormal O=Fe=O species on Fe–N_2_C_2_, MS6: adsorption of C_6_H_6_, TS2: transition state of C–O bond generation, MS7: C_6_H_6_O adsorption structure, TS3: transition state of H transfer from C to O, MS8: product bonded species, FS: regeneration of activity Fe=O center). The white, gray, red, blue and bluish violet balls refer to H, C, O, N, and Fe atoms, respectively.

Pan *et al.*^24^ further revealed the intrinsic electronic properties of the coordination effect through DFT calculations, as shown in Fig. 5. The coordination mode of Fe–N leads to a change in the structure and electronic properties of the catalyst, thus affecting the path of the catalytic reaction and the formation of intermediates. The increase in the Fe–N coordination number facilitates the generation and activation of the crucial intermediate O=Fe=O species, thereby enhancing the BOR activity. More interestingly, when the coordinated N atom is replaced with one or two C atoms, the BOR activity of the monatomic Fe site catalyst decreases gradually, showing obvious coordination sensitivity. Therefore, the monatomic Fe center fixed by four nitrogen atoms showed the highest BOR performance, with 78.4% conversion and 100% phenol selectivity at 30 °C, exceeding those of all reported BOR catalysts.

The ORR activity with typical Fe–N_4_ active sites was improved by introducing suitable carriers. For example, the intrinsic ORR activity of the original Fe phthalocyanine (FePc) catalyst with the added two-dimensional (2D) material Ti_3_C_2_T_*x*_ as the carrier was twice as high as that of the FePc catalyst.^25^ FeN_2_ and FeN_6_ active sites are considered to have great potential in the development of new advanced Fe-based monatomic electrocatalysts. Yansong Zhu^26^ confirmed that the active site of a six-coordinated Fe(iii) complex [Fe(iii) (porphyrin)(pyridine)_2_] was six-coordinated FeN_6_. The non-noble metal catalyst for ORRs showed high activity and good durability. Zheng *et al.*^27^ designed and developed a range of molecule-level graphitic carbon nitride (g-C_3_N_4_)-coordinated transition metals catalysts for these oxygen electrode reactions. The correlation of experimental and computational results confirmed that this high activity originated from the precise M–N_2_ coordination in the g-C_3_N_4_ matrix.

**Fig. 6:**
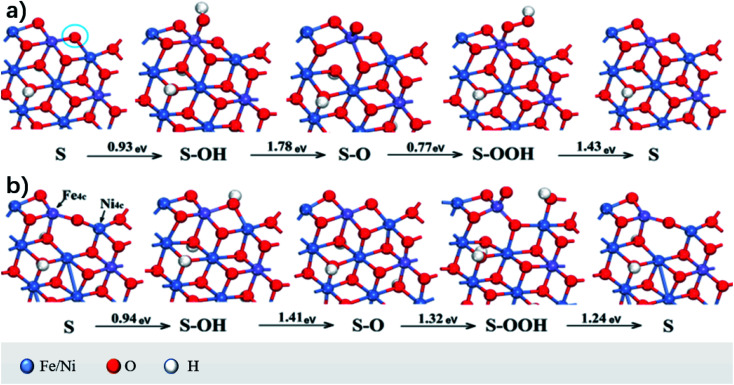
OVs' effect on OER catalysis performance predicted by DFT calculations. Intermediate states (S represents the active site on the surface of the catalyst, S–OH, S–O, and S–OOH, four elementary reactions: ① S + OH^−^ → S–OH + e^−^, ② S–OH + OH^−^ → S–O + H_2_O + e^−^, ③ S–O + OH^−^ → S–OOH + e^−^, ④ S–OOH + OH^−^ → S + O_2_ + H_2_O + e^−^) and Gibbs free energy changes associated with the elementary reactions of OERs on Fe-doped NiOOH (a) without OV and (b) with OV.

Asnavandi *et al.*^28^ first studied the overpotential of the surface without oxygen vacancies (OVs). Oxygen vacancies refer to vacancies formed in metal oxides or other oxygen-containing compounds where oxygen atoms (oxygen ions) in the crystal lattice break away, resulting in oxygen deficiency. Fig. 6a shows the geometric structure of the OER and the calculated Δ*G*. Δ*G*_max_ = 1.78 eV, which led to an overpotential of 0.55 V when the OER occurred at the Fe site. When oxygen (represented by the circle in Fig. 6b) was removed to produce OV and the low coordination metals (LCMs) were marked as Fe 4c and Ni 4c, the reactant species (O, OH, and OOH) got adsorbed and relaxed completely on it. Its geometric structure and calculated value of Δ*G* are shown in Fig. 6b; Δ*G*_max_ is 1.41 eV and overpotential *η* = 0.18 V. Compared to the case without OV, the overpotential decreased by 0.37 V, which indicated that the LCM activity associated with OV was higher, which is consistent with the understanding in the earlier study that the high catalytic performance of NiFe–OOH results from poorly coordinated metals such as defects, steps, and edges.^29^

In order to further describe the active sites and catalytic performance of Fe monoatomic catalysts, we collected some information about Fe monoatomic catalysts, including reaction precursors, types of active sites, and loading, as presented in Table 2. These are the key factors affecting its activity. Therefore, the activity of the catalyst can be regulated by selecting precursors or loading to form Fe active sites. For example, doping heteroatoms (such as P, S, and N) on the substrate aids in the adjustment of the electronic structure of the Fe active site. It is also possible to control the density of Fe active points by changing the Fe loading rate, thereby adjusting the performance and application of the catalyst.

**Table 2:** Brief introduction of some information on Fe-SACs

Sample	Active site	Precursor	Loading rate	Application	Reference
S,N–Fe/N/C–CNT	Fe–N_*x*_	FeCl_3_, KSCN, carbon nanotubes, 2,2′-bipyridine		ORR, OER	16
Fe–NC SAC	Fe–N_4_	Fe (NO_3_)_3_, glucose, oxygen-rich porous carbon, melamine	12.1wt%	CO2RR	17
Fe–N_*x*_–C	Fe–N_*x*_	FeSO_4_, Phen, ZIF-8	0.81wt%	ORR	18
CNT/PC	Fe–N_4_	Fe(iii)TMPPCl, PC, carbon nanotubes	2.9wt%	ORR	30
Fe/N–GPC	Fe–N_4_	FeCl3, dicyandiamide, MIL-101-NH_2_	1.1wt%	ORR	31
Fe SAS/N–C	Fe–N_4_	PEI, Phen, FeCl_2_, C3N4 nano sheet	3.5wt%	ORR	21
Fe SAs–N/C-20	Fe–N_4_	Fe Pc, ZIF-8	0.20wt%	ORR	22
Fe–CBDZ	Fe–N_*x*_	Fe (NO_3_)_3_·9H_2_O, CBDZ		ORR	23
Fe–N–C	Fe–N_4_	Fe^3+^, ZIF-8	1.5wt%	ORR	32
Fe–N–C-950	Fe–N_4_	Fe (NO_3_) _3_, ZIF-8	0.137wt%	ORR	33
Fe Pc/Ti3C2Tx	Fe–N_4_	Fe Pc, Ti_3_C_2_T_*x*_	4.09wt%	ORR	25
PpPD–Fe–C	Fe–N_6_	*p*-Phenylenediamine, ferric chloride, carbon black	12.08wt%	ORR	26
Fe^3+^–N–C	Fe–N_*x*_	FeCl_2_, ZIF-8	2.6wt%	CO2RR	34
Fe/NG-750	Fe–N_4_	Graphene oxide, FeCl_3_	0.52wt%	CO2RR	20
ISAS–Fe/NC	Fe–N_4_	C_34_H_32_FeN_4_O_4_, ZIF-8	4.2wt%	NRR	19
Fe1–N–C	Fe–N_4_	FeCl_2_·4H_2_O, meso-tetra (4-carboxyphenyl) porphine tetramethyl ester	4.51wt%	NRR	35
Fe–N–C	Fe–N_*x*_	Glucose, dicyandiamide, FeCl_2_·4H_2_O	0.26wt%	ECL	36
Fe/GD		Graphdiyne, FeCl_3_	0.680wt%	HER	8

In short, the main factors affecting the catalytic activity of Fe-SACs and its potential application in electrocatalysis include the coordination structure and electronic structure of Fe, the morphology and electronic structure of the conductive matrix, and the total number of Fe atom active sites.

## 3. Synthetic strategies of Fe-SACs

Recently, various experimental techniques for the preparation of Fe single atoms have been reported. Moreover, according to the reported process, the preparation of Fe monoatomic catalysts can be summarized into the following three strategies.

### 3.1. Strategy of wrapping iron active sites in roasted metal–organic frameworks

**Fig. 7:**
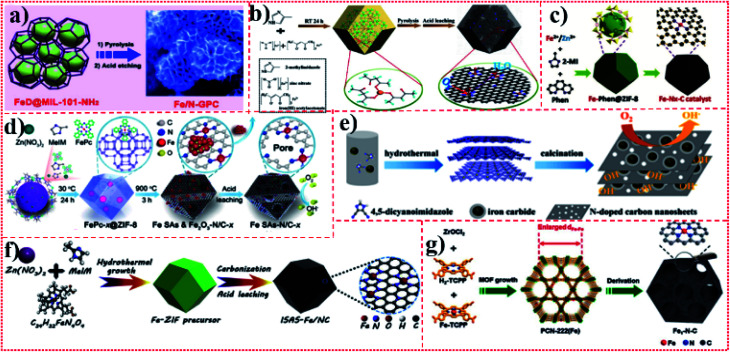
(a)–(g) Schematic of Fe-SACs with a metal–organic framework as a catalytic application platform.

In recent years, metal–organic frameworks (MOFs) have attracted significant attention because of their good porous structure and highly ordered arrangement of organic connectors and metal nodes. Both MOFs and MOF-derived materials can still maintain their specific shape and pore structure after pyrolysis at high temperatures. Notably, they provide a pathway for the effective transfer and diffusion of substrates and products, providing a high-quality platform for Fe active sites. The synergistic effect and pore limitation effect can be produced between the active site of Fe and the MOF shell. Metal nodes and aromatic connectors in MOFs can establish charge transfer interactions with Fe active sites by coordination or π–π interaction, thus performing collective functions compared to single-component materials. Moreover, the MOF shell effectively wraps the Fe active site and prevents the agglomeration and leaching of Fe atoms, thus improving the stability of the catalytic process. Therefore, MOF coating strategy is widely used in the preparation of Fe-SACs. However, although intensive efforts and great progress have been made in this area, the study on Fe active sites coated with porous MOFs is still in its infancy and many challenges still need to be overcome. The composition and spatial distribution of Fe active sites in MOFs are still difficult to control, and it is also very hard and challenging to effectively balance the relationship between heterogeneous nucleation and controllable growth of MOFs around Fe active sites and self-nucleation and homogeneous growth. Moreover, the electrical conductivity and ionic conductivity of MOFs are also important factors significantly affecting its electrocatalytic performance. For example, Qi-Long Zhu and his colleagues^31^ used MIL-101-NH_2_ as the MOF host to accommodate dicyandiamide, the nitrogen source with high nitrogen content, and FeCl_3_, the most readily available Fe source. In order to avoid the deposition of precursors on the outer surface of a MIL-101-NH_2_ crystal, dicyandiamide and FeCl_3_ were quantitatively coated by a double-solvent method. Fig. 7a shows the monatomic catalyst, Fe/N-GPC, obtained by pyrolysis and acid etching of the synthesized MOF composite, exhibiting that the complete MOF skeleton of the catalyst was well maintained. Xiao *et al.*^33^ pyrolyzed Fe triacetylacetonate Fe-doped ZIF-8 as the metal source, nitrogen source, and carbon source to form monoatomic catalyst Fe–N–C-950 (Fig. 7b). Han *et al.*^18^ synthesized Fe–Phen complexes at room temperature using FeSO_4_ and Phen as non-precious metal and organic ligands, respectively. Then, during the growth of ZIF-8, the Fe–Phen species were wrapped in nano-cages. The monoatomic catalyst Fe–N_*x*_–C was obtained after pyrolysis in an argon atmosphere at 900 °C (Fig. 7c). Jiang *et al.*^22^ used ZIF-8 as the host of MOFs to hold Fe(ii)Pc. During the assembly of Zn(ii) and 2-methylimidazole, Fe(ii)Pc molecules were encapsulated in the cavity of a zeolite imidazole skeleton to form FePc-*x*@zif-8 nanocomposites. Monatomic catalyst Fe SAs–N/C-20 was obtained by pyrolysis and acid etching, and the complete MOF of the catalyst was well maintained (Fig. 7d). Li *et al.*^37^ synthesized a flaky MOF by the interaction between N-rich dicyanoimidazole and ferric acetate. By using the MOF as the host, Fe acetate was coated, and the monoatomic catalyst DCI–Fe was prepared by pyrolysis (Fig. 7e). Lü *et al.*^19^ prepared a bimetallic Fe/Zn zeolite imidazole framework (ZIF-8) by a hydrothermal method and then carbonized and etched it to obtain monoatomic catalyst ISAS–Fe/NC (Fig. 7f). Zhang *et al.*^35^ synthesized a Fe-containing rod-like MOF from ZrOCl_2_ and tetra(*p*-carboxyphenyl)porphyrin and tetra(*p*-carboxyphenyl)porphyrin Fe in DMF and trifluoroacetic acid. In this porphyrin MOF, they successfully constructed the Fe atom-dispersed catalyst Fe1–N–C by reasonably controlling the distance between the adjacent Fe atoms (Fig. 7g). Zhang *et al.* prepared catalyst precursors with different Fe contents by using Fe ions to replace part of the original Zn ions to control Fe doping in ZIF-8. Through the subsequent high-temperature treatment, the Fe-doped ZIF was directly converted into carbon with high specific surface area (SSA) to form a specific framework of the Fe-based porous carbon monoatomic catalyst Fe-ZIF.^6,32,34,38^

### 3.2. Strategy including modified carbon-rich carriers

**Fig. 8:**
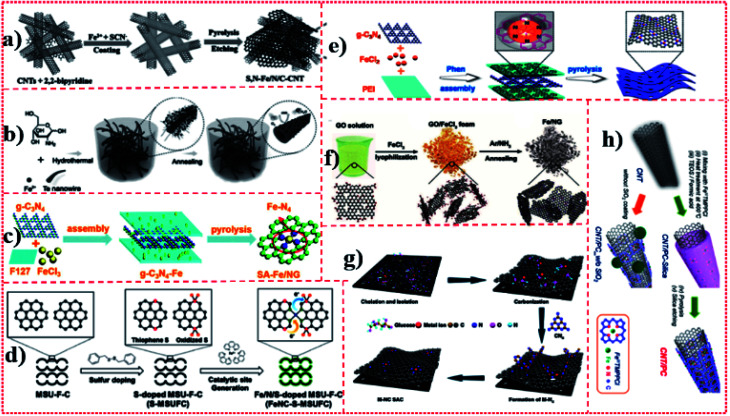
(a)–(h) Schematic of Fe-SACs for the synthesis of the carbon matrix and metal complex hybrid.

Carbon-rich carriers including graphene (or graphene oxide (GO)) sheets, carbon nanotubes (CNTs), carbon nanofibers, carbon nanospheres, nano-carbon derived from polycarbonate, and other polymer-derived nano-carbon materials have porous structures for rapid mass and energy transfer and an adjustable chemical coordination environment, thus they can be considered as ideal carriers for Fe monoatomic catalysts. The use of chitosan, cyanamide, dicyandiamide, melamine, and porphyrin also shows the potential to capture Fe atoms *via* surface modification of carbon-rich carriers, in order to achieve efficient electrocatalytic performance. Although this strategy has been developed to some extent, it still faces multiple challenges to be addressed. The loading of this type of Fe monatomic catalyst is relatively low, which may limit its further development in large-scale applications. The carrier (graphene or GO) mainly depends on the structure of graphene or GO, which is fragile and easy to break, thus it is difficult to be characterized and controlled by a synthesis strategy. This method of preparing Fe active site by introducing doping and manufacturing defects to adjust the local electronic state on the surface of the carrier is difficult to control regularly. Chen *et al.*^16^ achieved monoatomic dispersion of Fe(S,N–Fe/N/C-CNT) on the layered carbon modified with N and S by coating 2-bipyridine and Fe salt precursors onto the surface of CNTs, followed by pyrolysis and acid etching in a nitrogen atmosphere (Fig. 8a). Zhu *et al.*^39^ prepared CNT precursors by a one-step hydrothermal method using tellurium nanowires, glucosamine hydrochloride, and ammonium ferric sulfate (catalyst) at 180 °C for 15 h. The resulting hydrogel was soaked several times in distilled water to remove impurities and then freeze-dried. Finally, the monatomic catalyst Fe–N-CNTAS-5-900 was obtained by heat treatment in a nitrogen atmosphere for 2 h (Fig. 8b). Yang *et al.*^13^ used Fe-loaded F127 [F127 is a water-soluble surfactant of polyoxyethylene–polyoxypropylene–polyoxyethylene (PEO–PPO–PEO)] and g-C_3_N_4_ to form layered precursors, and then obtained monoatomic catalyst SA-Fe/NG by pyrolysis (Fig. 8c). Further, Mun *et al.*^40^ used ferrous chloride hexahydrate and 1,10-phenanthroline (Phen) as precursors and mesoporous carbon foam as a support, and obtained monoatomic catalyst FeNC–S-MSUFC by pyrolysis and acid etching (Fig. 8d). Yang *et al.*^21^ used polyetherimide (PEI) polymers as carbon precursors and Phen ligands as spatial isolating agents for Fe ions to promote their complete conversion into a single Fe atom without forming Fe nanoparticles (NPs), and N-doped porous carbon nanosheets as carriers to form a highly stable monoatomic catalyst, Fe SAs/N–C, by high-temperature pyrolysis (Fig. 8e). Zhang *et al.*^20^ used GO as the precursor, annealed the mixture of GO and FeCl_3_ in an Ar/NH_3_ atmosphere at 800 °C and fixed the Fe atom on graphene by a N bond, and obtained Fe/NG with monoatoms dispersed on NG (Fig. 8f). Zhao *et al.*^17^ reported a cascade anchoring strategy for synthesizing Fe–NC structures. Fe ions were first chelated with chelating agents (such as glucose), then fixed on oxygen-rich porous carbon carriers with high SSA, and finally pyrolyzed at a certain temperature to form Fe–N_*x*_ catalytic sites. The function of the chelating agent is to protect the Fe ion and combine it with the oxygen-rich carbon carrier (Fig. 8g). Sa *et al.*^41^ mixed CNTs with porphyrin Fe precursors, heated the CNTs adsorbed by porphyrin to 400 °C, and formed CNTs with a porphyrin layer *via* π–π interaction. Second, the surface of the composite was covered with a layer of silicon dioxide (SiO_2_). Finally, the ternary composite was pyrolyzed at high temperatures, and then the SiO_2_ layer was etched to obtain the Fe monoatomic catalyst CNT/PC (Fig. 8h). Varela *et al.*^42^ first carried out *in situ* polymerization using Fe chloride and aniline as precursors and ammonium persulfate as an oxidant, and then added Kaijin black carbon powder for continuous annealing and acid leaching cycles to synthesize the monatomic Fe-doped N porous carbon black catalyst Fe–N–C. Choi *et al.*^43^ prepared monoatomic catalyst Fe–N–C by pyrolysis and acid etching using ferric acetate and Phen as precursors and ZIF-8 as a carrier. Jiang *et al.*^44^ coated CNTs with glucose in the presence of Fe salt precursors, and then prepared monoatomic catalyst Fe@C–FeNC by pyrolysis in the presence of melamine.

### 3.3. Strategy for *in situ* preparation of small molecule precursors

**Fig. 9:**
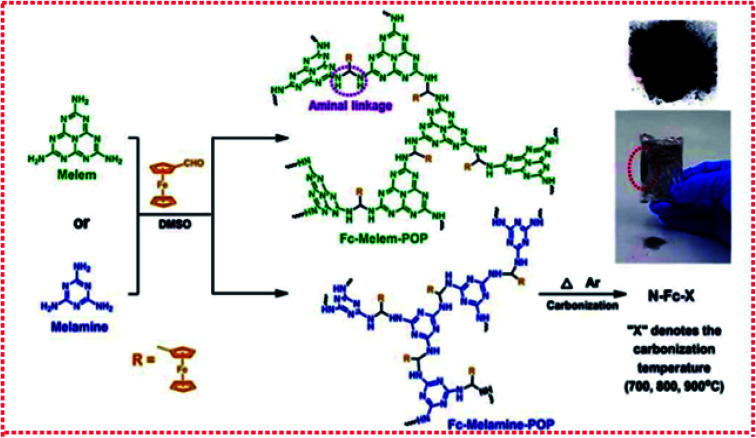
Synthetic route for the preparation of ferrocene-based POPs and nitrogen-doped carbon catalysts.

The preparation process of Fe-SACs using an *in situ* strategy is relatively simple, can control the dispersion of Fe atoms, and easily used on a large scale. However, the small molecule precursors usually have a relatively low thermal decomposition temperature, which makes the carbonization of precursors difficult. Therefore, appropriate selection of small molecule precursors is very important. Thus far, most of the precursors are nitrogen-rich molecules. Moreover, the catalyst prepared by this method is also difficult to have a consistent geometric morphology, and the selectivity of the catalysts may also be poor. For example, Zhou *et al.*^45^ easily synthesized novel low-cost ferrocene-based porous organic polymers (POPs) by the Schiff base reaction between melamine and ferrocene aldehyde without any catalyst. Through the direct carbonization of the synthesized ferrocene-based porous organic polymer, the transformation from amorphous ferrocene-based POP to the crystalline Fe_3_C/Fe carbon composite was further realized to prepare monoatomic catalyst N-FC-800 (Fig. 9). Gu *et al.*^36^ mixed glucose, dicyandiamide, and ferrous chloride tetrahydrate in an aqueous solution and then carried out freeze–drying and pyrolysis to obtain monoatomic catalyst Fe–N–C. Serov *et al.*^23^ prepared monoatomic catalyst Fe–CBDZ by pyrolysis and acid etching using ferric nitrate and carbendazim as precursors and SiO_2_ powder as a template. Owing to the elimination of artificial carbon in the material design, the unique process method, and the reasonable selection of precursors based on high carbon content, high N/C ratio and low volatile content, the catalyst showed a higher active site density and a higher mass activity.

## 4. Electrochemical applications of iron-based single-atom catalysts

The development of efficient, durable, and economical non-precious metal monatomic electrocatalysts is of great significance to the development and commercial application of electrochemical energy conversion technology; however, this is still a huge challenge. In recent years, Fe-SACs used in various reactions have been developed, which have excellent activity, selectivity, and stability. In the following chapters, the electrochemical applications of Fe-based monatomic catalysts in key energy conversion reactions such as ORRs and CO_2_RRs are introduced and summarized, in order to demonstrate the great potential of Fe-based monatomic catalysts in achieving efficient and selective electrochemical processes.

### 4.1. Oxygen reduction reaction

Environmentally friendly energy storage process/device/technology that converts chemical energy into electricity through devices such as fuel cells and metal–air cells is essential to reduce pollution and the greenhouse effect because the world needs to shift from carbon and fossil fuel (coal, oil, and natural gas)-driven economy to more sustainable and green options.^46,47^ However, one of the main bottlenecks in the commercialization of these devices is the requirement of ORRs in their operation, because of their high overpotential due to the slow cathodic kinetics.^48^ Fe-based monatomic catalysts are considered to be one of the most important (ORR) catalysts for oxygen reduction. They are potential substitutes for Pt-based catalysts; therefore, they have a bright future in the development of non-noble metal-based catalysts. At present, according to the different products, the path of the ORR can be divided into the following two types: one is the four-electron (4e) reaction mechanism of complete reduction, under acidic (or alkaline) conditions, in which oxygen is reduced to water (or OH^−^); and the other is a partial reduction of two-electron (2e) reaction mechanism. Under acidic (or alkaline) conditions, oxygen is reduced to hydrogen peroxide (or HO^2−^). ORRs of two paths in different electrolytes are shown in Table 3.^49^ In the fuel cell process, the 4e path is preferred because it provides high current efficiency, while the 2e reduction pathway is used in the industrial production of H_2_O_2_. Therefore, 4e complete reduction of ORR catalyst is required when selecting the ORR catalyst.^50^

**Table 3:** Electrode reaction of oxygen reduction reactions in different electrolytes

Reaction path	Electrolyte solution	Reaction equation
4e	H^+^	O_2_ + 4H^+^ + 4e^−^ = 2H_2_O
	OH^−^	O_2_ + 2H_2_O + 4e^−^ = 4OH^−^
2e	H^+^	O_2_ + 2H^+^ + 2e^−^ = H_2_O_2_
	OH^−^	O_2_ + H_2_O + 2e^−^ = HO_2_^−^ + OH^−^

**Fig. 10:**
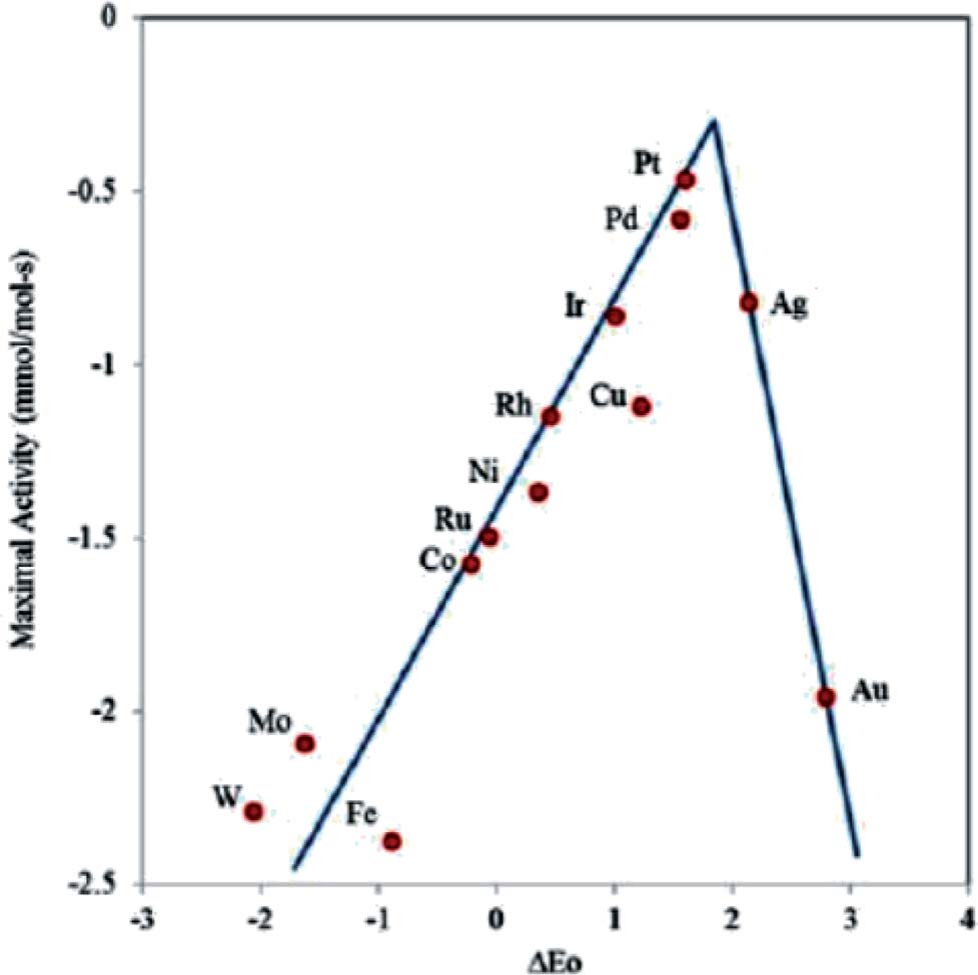
Volcano plot showing the relationship between the oxygen binding energy and the maximal activity calculated using the Sabatier analysis.

Fig. 10 shows the construction of a “volcano map” based on theoretical calculation and experimental data.^51^ Although Fe is distributed at the bottom of the volcanic map, the activity of ORR can be improved by regulating the metal-carrier interaction and promoting the adsorption of key intermediate systems to reduce the Gibbs free energy of ORRs. Some Fe-based monatomic catalysts reported in recent years have excellent ORR catalytic activity, even exceeding that of the commercial Pt/C catalysts at the top of the “volcano map”.

**Fig. 11:**
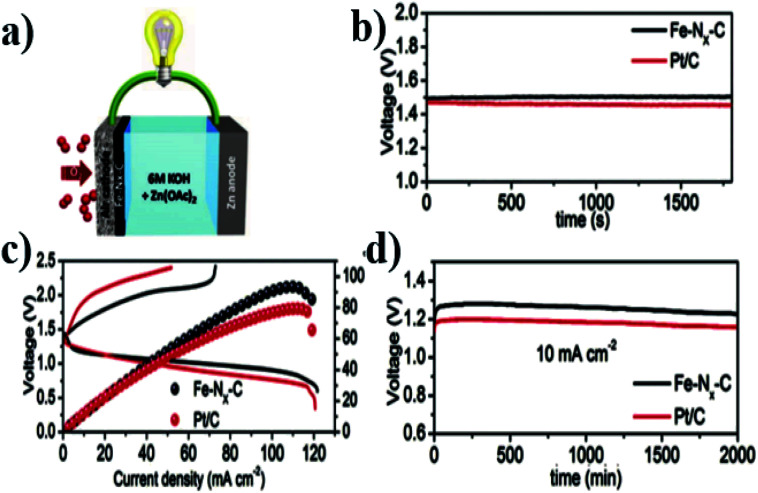
(a) Schematic showing the structure of ZAB. (b) OCV plots of the liquid ZABs. (c) Discharging and charging polarization curves and the corresponding power density plots. (d) Long-term discharging performance of primary ZABs.

**Fig. 12:**
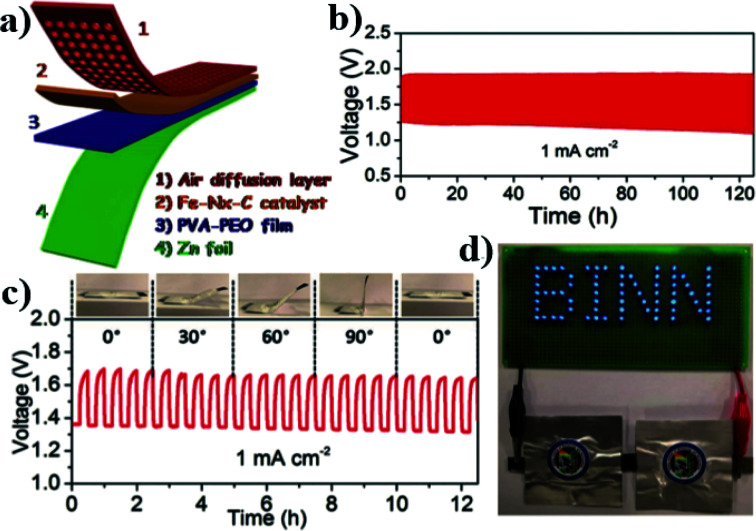
(a) Schematic of all-solid-state ZAB. (b) Cycling performance of the all-solid-state ZAB tested at a current density of 1 mA cm^−2^. (c) Foldability test of all-solid-state ZAB bended in different angles from 0° to 90° at a current density of 1 mA cm^−2^. (d) A lighted LED array powered by two all-solid-state ZABs in series.

**Fig. 13:**
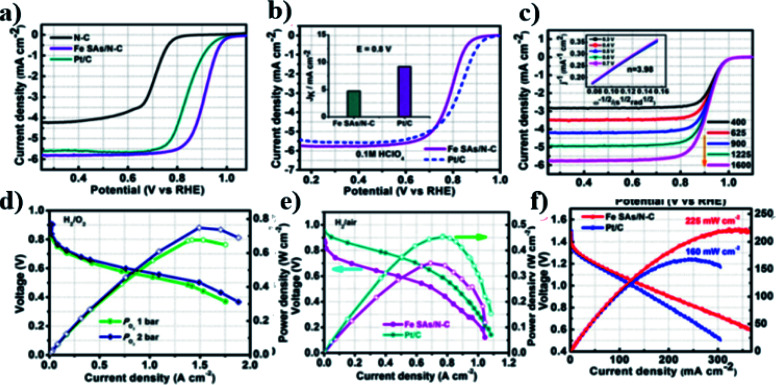
(a) LSV curves of N–C, Fe SAs/N–C and Pt/C catalysts in O_2_ saturated 0.1 M KOH. (b) LSV curves of Fe SAs/N–C and Pt/C catalysts in 0.1 M HClO_4_. (c) LSV curves of Fe SAs/N–C at different rotation rates. Inset: *K*–*L* plots. (d) H_2_/O_2_ PEMFC performance of Fe SAs/N–C-based MEA. (e) H_2_/air PEMFCs performances using Fe SAs/N–C and Pt/C as cathode catalysts. (f) Discharge curves and the corresponding power density plots with Fe SAs/N–C and Pt/C as cathode catalysts.

The atomically dispersed Fe–N_*x*_–C catalysts prepared by Han *et al.*^18^ showed ultra-high ORR activity, stability, and methanol resistance, and exhibited better performance than most Pt-free catalysts and industrial Pt/C catalysts in an alkaline electrolyte. The half-wave potential of ORRs of the catalyst was 0.91 V, which is higher than that of commercial Pt/C (0.82 V). The primary zinc–air battery with the Pt/C cathode catalyst showed excellent electrochemical performance with open-circuit voltage (OCV) as high as 1.51 V and power density as high as 96.4 mW cm^−2^. The rechargeable zinc–air battery with the Fe–N_*x*_–C catalyst and an alkaline electrolyte showed excellent cycle performance within 300 h, and the initial round-trip efficiency was 59.6%, as shown in Fig. 11a–d. Moreover, the rechargeable all-solid-state ZABS with Fe–N_*x*_–C catalyst has a high OCV of 1.49 V, a long cycle life of 120 h, and foldability as shown in Fig. 12a–d. Zhu *et al.*^31^ prepared electrode using the Fe/N-GPC catalyst, which exhibited high catalytic activity with an initial potential of 0.85 V and a half-wave potential of 0.63 V. The electron transfer number of Fe/N-GPC was calculated to be 3.62–3.73 at 0.4–0.2 V, suggesting that the Fe/N-GPC electrode also mainly favored an efficient four-electron transfer process in acidic media, showing great application potential. The catalyst FeSAs/N–C electrode prepared by Yang *et al.*^21^ showed high ORR catalytic activity. Under alkaline conditions, the initial potential was 1.02 V and the half-wave potential was 0.91 V (Fig. 13a), while under acidic conditions, the initial potential was 0.95 V and the half-wave potential was 0.798 V (Fig. 13b). The electron transfer number of the FeSAs/N–C electrode was found to be 3.98 (Fig. 13c), indicating that the mechanism reaction of the FeSAs/N–C electrode is mainly 4e transfer process in an acidic or alkaline medium. The FeSAs/N–C catalyst is used as the cathode of proton exchange membrane fuel cell (PEMFC). At 0.1 and 0.2 MPa back pressure, the maximum power densities of FeSAs/N–C-based H_2_/O_2_ PEMFC were found to be 0.68 and 0.75 W cm^−2^, respectively (Fig. 13d). When using this FeSAs/N–C catalyst for H_2_/air PEMFC, this single PEMFC exhibited a peak power density of 0.35 W cm^−2^, and the maximum power density of the Zn–air battery using the FeSAs/N–C catalyst was as high as 225 mW cm^−2^ (Fig. 13e and f), indicating that FeSAs/N–C is an excellent Pt-free catalyst.

**Fig. 14:**
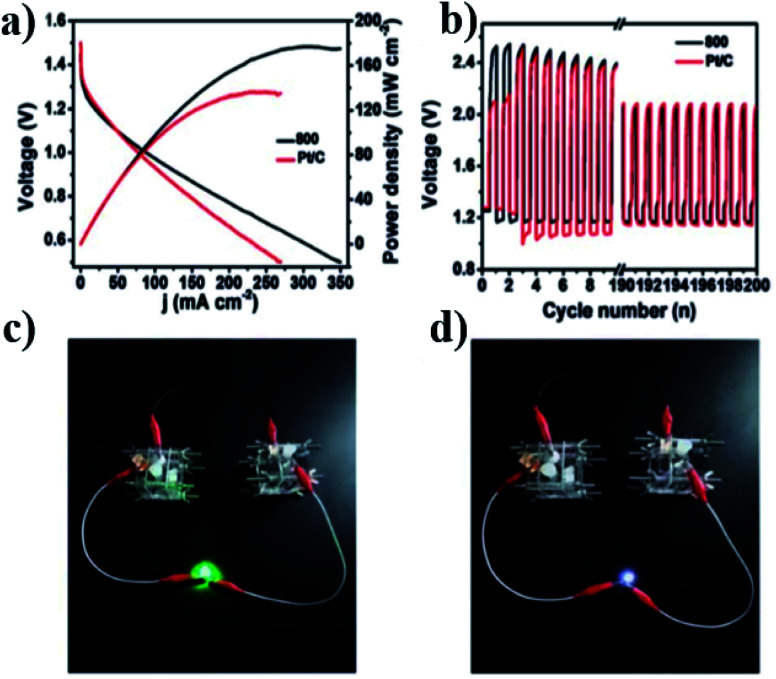
(a) Discharge polarization curves and the corresponding power density curves of primary zinc–air batteries using the N-FC-800 as air catalysts (mass loading of 1 mg cm^−2^); (b) cycling performance of rechargeable zinc–air batteries at a current density of 10 mA cm^−2^; (c and d) photographic images of a two-electrode rechargeable zinc–air battery lighting up a LED light (blue for (c) and white for (d)).

Zhou *et al.*^45^ prepared the catalyst (N-FC-800), which showed high initial potential and half-wave potential of 0.96 and 0.82 V, respectively, exhibiting good activity. Based on this, a rechargeable zinc–air battery was assembled using N-FC-800 as the positive catalyst. Compared to commercial Pt/C, the N-FC-800 battery has a higher power density of 178 MW ± cm^2^ and a smaller charge–discharge voltage gap of 0.94 V (Fig. 14a), good stability, and less activity attenuation (1.0%) in long charge–discharge cycles (Fig. 14b), and it successfully lit the LED lamp (Fig. 14c and d).

### 4.2. Oxygen evolution reactions

The OER is the reverse reaction process of ORRs, that is, the oxidation of H_2_O to O_2_ is the reaction of the air electrode in the metal–air battery during the charging process. In the past few decades, electrocatalytic OERs have received extensive attention globally, and a large number of catalysts have been developed to improve the kinetics and stability of OER electrodes in different electrolytic environments. Among them, rutile RuO_2_ and IrO_2_ show good OER performance, and they are used as reference catalysts for research on OER performance.^52,53^

Nonetheless, they are prone to further oxidation at high potentials, resulting in their instability at high potentials. More importantly, they are all precious metal catalysts, and their high costs are not suitable for large-scale production applications. Owing to the rapid development of monatomic catalysts in recent years, it has been found that Fe-based monatomic catalysts also have good OER activity. For example, Junxing Han^18^ prepared the Fe–N_*x*_–C catalyst and compared it with the most advanced OER catalyst RuO_2_. The overpotential of the Fe–N_*x*_–C catalyst was found to be 600 mV when the current density was 10 mA cm^−2^ (marked as *E*_*j*10_), which is close to the overpotential of RuO_2_ (530 mV). Potential difference Δ*E* (Δ*E* = *E*_*j*10_ − *E*_1/2_) is an important parameter to evaluate the redox catalytic performance of electrocatalysts. The Δ*E* value of the Fe–N_*x*_–C catalyst is about 0.92 V, which is better than that of RuO_2_ for OERs (*E*_*j*10_) (Δ*E* = 0.94 V). Guoqiang Shen^54^ report a facile adsorption–oxidation strategy to anchor Fe(iii) atomically on ultrathin TiO_2_ nanobelt to synergistically lower the spin state (e_g_ filling ∼1.08) to enhance the adsorption with oxygen-containing intermediates and improve the electro-conductibility for lower ohmic loss. The electronic structure of the catalyst is predicted by DFT calculations and perfectly confirmed by experimental results. The catalyst exhibits superior performance for OERs with an overpotential 270 mV @ 10 mA cm^−2^ and 376 mV @ 100 mA cm^−2^ in an alkaline medium, which is much better than that of IrO_2_/C and RuO_2_/C.

### 4.3. Carbon dioxide reduction reactions

The electrochemical reduction of CO_2_ to CO is a 2e/proton reaction, which includes two basic steps.^55–57^ CO_2_ is first adsorbed on the surface of the catalyst, and one electron is transferred to the adsorbed CO_2_ to generate surface-adsorbed CO_2_^−^; the subsequent combination of CO_2_^−^ and a proton occurs to form *COOH, where * represents the adsorbed substance on the surface. Then, *COOH reacts with protons to remove H_2_O to form * CO intermediates. Finally, the free gaseous CO was formed by peeling off the surface of the catalyst. Based on this reaction path, an ideal catalyst needs to have a suitable adsorption energy for the main intermediates, which is beneficial to promote the activation of CO_2_ to form *COOH and the desorption step of *CO. The electrochemical reduction of CO_2_ to CO provides a promising pathway to reduce CO_2_ emissions and increase the value of chemical fuel to alleviate the energy crisis. Therefore, development of efficient and selective CO_2_RR electrocatalysts is of great significance. Fe-based monatomic catalysts exhibit the highest atomic efficiency and low coordination metal sites and show excellent activity in various reactions. Compared with the nano-cluster or nano-particle catalyst, the monatomic catalyst has a relatively uniform active center and geometric configuration, which gives the reaction substrate a similar environment, which is very beneficial to improve the catalytic selectivity. Therefore, Fe monatomic catalysts show great hope in achieving high efficiency and selectivity in the CO_2_RR process. Gu *et al.*^34^ prepared a catalyst Fe^3+^–N–C consisting of dispersed monatomic Fe sites, which could produce CO at an overpotential as low as 80 mV. At an overpotential of 340 mV, the local current density reached 94 mA cm^−3^. The active sites of the catalyst were discrete Fe^3+^ ions, which coordinated with pyrrole nitrogen (N) atoms on N-doped carbon carriers and maintained +3 oxidation states in the process of electrocatalysis, which was possibly achieved by electronic coupling with conductive carbon carriers. Electrochemical data show that compared to the traditional Fe^2+^ site, the Fe^3+^ site undergoes faster CO_2_ adsorption and weaker CO absorption, thus obtaining better activity. Huan *et al.*^58^ prepared Fe_0.5_d material as a catalyst with the highest activity and the best selectivity, which could produce CO with high FY (up to 90%) and low overpotential (190 mV overpotential relative to the equilibrium potential of CO_2_/CO in 0.5 M NaHCO_3_ aqueous solution) and could be electrolyzed for a long time. Interestingly, the selectivity of these materials for CO_2_ reduction depends on the ratio of isolated FeN_4_ sites to Fe-based NPs, and the higher the ratio, the better the selectivity. These positions are reminiscent of soluble Fe porphyrins, and they have been proved to be excellent catalysts for the conversion of CO_2_ to CO. Zhang *et al.*^16^ synthesized dispersed Fe atoms on nitrogen-doped graphene (Fe/NG) as efficient electrocatalysts for the reduction of CO_2_ to CO. The reduction overpotential of Fe/NG was low, and the Faraday efficiency was as high as 80%. The evidence of structural analysis and control experiments of the catalyst system exhibited that the isolated Fe–N_4_ structure was the main reason for the reduction of CO_2_ to CO. Some Fe–N–C catalysts composed of uniformly dispersed Fe–N_*x*_ sites showed impressive high activity and remarkable selectivity for CO and hydrocarbons. In the future industrial CO_2_ gas diffusion cathode (CCCS), these catalysts can replace carbon-supported Au catalysts for the reaction of CO_2_RR to CO/H_2_ gas mixtures.^42,59^

### 4.4. Nitrogen reduction reactions

The traditional Haber–Bosch nitrogen production process requires high temperatures, high pressures, *etc.*, and the ammonia synthesis process usually requires about 485 kJ mol^−1^ energy input.^60^ Clearly, this is large energy consumption, and thus, ways to reduce this requirement are also highly desirable. Moreover, subsequent discussion also confirms that this is actually a very challenging goal. Under this background, electrochemical reduction of nitrogen to ammonia is considered as a promising alternative to the energy- and capital-intensive Haber–Bosch process; thus, it has attracted significant attention in the scientific community. The basic equation of the process can be expressed as follows:^61^

Anode (acid condition)
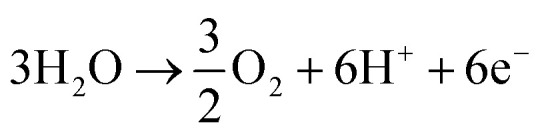
Anode (alkaline condition)
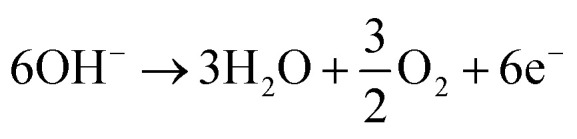
Cathode (acid condition)N_2_ + 6H^+^ + 6e^−^ → 2NH_3_Cathode (alkaline condition)N_2_ + 6H_2_O + 6e^−^ → 2NH_3_ + 6OH^−^Overall
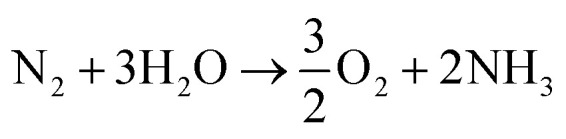


**Fig. 15:**
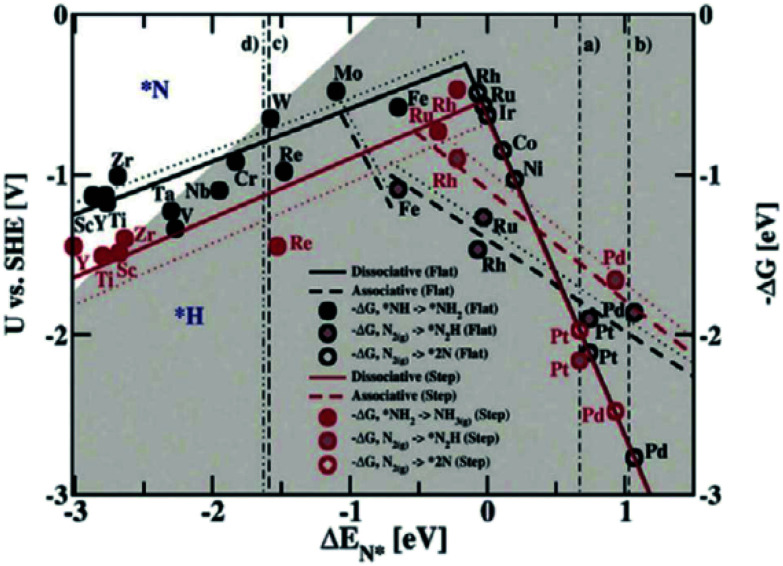
NRR volcano curve calculated by theory.

Norskov *et al.* studied the NRR performance of different metal catalysts by theoretical calculation (Fig. 15) to reveal the relationship between N_2_ adsorption and catalytic ammonia production. If only the activity of NRR is considered, the metals Mo, Fe, Ru, and Rh have been found to be more appropriate.^62^

In the past few years, Fe-based monatomic catalysts have been proposed as catalysts for NRRs, and they have shown very high performance in terms of FE, NH_3_ yield, and stability. The simplicity and uniformity of its structure are helpful for the comprehensive understanding of the relationship between structure and catalytic performance, which is helpful to achieve a reasonable catalyst design on the atomic scale. Lü *et al.*^19^ reported an Fe monoatomic catalyst (ISAS–Fe/NC) for electrochemical ammonia synthesis at room temperature. At −0.4 V (RHE), the catalyst exhibited high Faraday efficiency (18.6 ± 0.8%) and NH_3_ yield (62.9 ± 2.7 μg h^−1^ mg_cat._^−1^) in a neutral aqueous solution at room temperature. Moreover, the activity attenuation of the catalyst within 24 h of electrolysis was negligible. The atomically dispersed Fe–N_4_ structure could activate N_2_ molecules and act as the active site of NRRs. Rui Zhang *et al.*^63^ prepared monoatomic Fe ion-implanted nitrogen-doped carbon catalysts (Fe_1_–N–C) using iron-modified porphyrin metal–organic frameworks (MOFs) as precursors by a mixed coordination strategy. Benefiting from the highly dispersed single-atom Fe sites, hierarchically porous structure and good conductivity, Fe_1_–N–C shows a FE of 4.51% and an ammonia yield rate of 1.56 × 10^−11^ mol cm^−2^. Noteworthy, for single-atom electrocatalysis, several studies have reported unique M–N_*x*_ sites. Comprehensive understanding of the relationship between structure and catalytic performance is required, which may provide a new opportunity for the development of advanced monatomic site catalysts for the synthesis of NH_3_*via* NRRs.^64^

### 4.5. Others

In addition to their applications in the above-mentioned fields, iron monatomic catalysts have also attracted the attention of researchers in the fields of HERs, electrochemiluminescence (ECL) and BOR. For example, Yurui Xue^8^ anchored Fe atoms on diacetylene graphite by a simple electrochemical reduction method to obtain monoatomic catalyst Fe/GD. It exhibited excellent performance toward HERs, and the overpotential was 66 mV and the Tafel slope was 37.8 mV dec^−1^ at 10 mA cm^−2^. After 5000 cycles, the polarization curve of Fe/GD remained unchanged in current density and was much better than that of commercial Pt/C in terms of stability. Gu *et al.*^36^ for the first time used the Fe monatomic catalyst (Fe–N–C SACs) as an advanced co-reaction accelerator to directly reduce dissolved oxygen (O_2_) to reactive oxygen species (ROS). Owing to its unique electronic structure and catalytic activity, it could efficiently produce a large number of ROS. These ROS could react with luminol anions and significantly amplified the ECL emission of luminol. Pan *et al.*^24^ fabricated a Fe–N_4_SAs/N–C catalyst, which exhibited the highest BOR performance by the monoatomic Fe center anchored by four coordinated nitrogen atoms, with a benzene conversion of 78.4% and a phenol selectivity close to 100%. When the N atom in the Fe–N_4_SAs/N–C catalyst was replaced with one or two C atoms, the activity decreased gradually, and the low activity could be improved by increasing the Fe–N coordination number. The results indicated that the catalytic performance could be changed conveniently and effectively by adjusting the coordination environment of SACs.

## 5. Challenges and opportunities

In recent years, owing to the development of high-resolution characterization technology and theoretical simulation, reasonable progress has been made in elucidating the real catalytic system of Fe monatomic materials. The potential of Fe monatomic catalysts for electrocatalysis is obvious; however, there are still many scientific and technical challenges that need to be overcome. The research on Fe monoatomic materials for electrocatalysis is in the initial stage, and there are few reports available in the literature. Although some progress has been made in the research on Fe monoatomic materials for electrocatalysis, most of the studies are focused on the new preparation methods, and the performance is not outstanding. Fe monatomic materials consist of fewer active sites, resulting in only low current density, which is not suitable for practical applications. However, with the increase in the load of Fe atoms, it is inevitable to face the problem of migration and aggregation of Fe atoms to form NPs. At present, the activity of most reported Fe monatomic materials is mainly due to their Fe–N_4_ active sites; nonetheless, studies on Fe–N_2_, Fe–N_6_, and other active sites with the same potential have rarely been reported. For example, the ORR activity of verified Fe–N–C-based catalysts is mainly attributed to their Fe–N_4_ active sites. However, according to theoretical prediction, this is not the most active site, but Fe–N_2_.^65,66^ Furthermore, basic problems such as Fe–N coordination environment still exist.

When exploring the preparation of high-performance Fe-SACs with specific functions, significant attention should be paid to the following points: (1) In order to obtain efficient Fe-SACs, development of more effective preparation methods is required to accurately regulate the anchorage point and the surface of the support. Defect engineering strategies, such as vacancies, amorphous phases, and structural defects, are used to adjust the electronic structure of the catalyst to obtain better catalytic performance. (2) The main challenge of Fe-SACs is that the loading of Fe active sites is very low, and further addition of Fe inevitably leads to the formation of Fe NPs. It is thus necessary to find or develop porous carriers such as porous 2D materials and 0D materials with abundant accessible surfaces or vacancies that can carry more monatomic active sites. (3) Enhancing the electrical conductivity of Fe-SACs is also an important approach to improve its electrocatalytic activity, for example, it can be achieved by improving the graphitization of carbon-containing materials. (4) It is also essential to use the method of theoretical calculation to study the catalytic performance of the active site of Fe-SACs. Thus far, little work has been done in this area. Therefore, the use of theoretical calculation method to select suitable materials will also be of great significance. (5) For researchers who study Fe-SACs, the development of its application in new fields should be regarded as an important task.

Although several challenges are encountered, there is a reason to be hopeful for Fe monoatomic catalysts that can be used for electrocatalysis, because the progress of *in situ* and non-*in situ* characterization techniques and the development of theoretical simulation can provide support for the study of the catalytic mechanism of Fe monoatomic catalysts. With the continued research on Fe monatomic catalysts, its application in the field of electrocatalysis will be more mature and become the star of the catalyst research.

## Conflicts of interest

The authors declare no conflict of interest.

## Supplementary Material

## References

[cit1] Chen F., Jiang X., Zhang L., Lang R., Qiao B. (2018). Single-atom catalysis: Bridging the homo- and heterogeneous catalysis. Chin. J. Catal..

[cit2] Ji S., Li Y. (2020). Chemical Synthesis of Single Atomic Site Catalysts. Chem. Rev..

[cit3] Peng Y., Lu B., Chen S. (2018). Carbon-Supported Single Atom Catalysts for Electrochemical Energy Conversion and Storage. Adv. Mater..

[cit4] Jiang K., Siahrostami S., Akey A. J., Li Y., Lu Z., Lattimer J., Hu Y., Stokes C., Gangishetty M., Chen G., Zhou Y., Hill W., Cai W.-B., Bell D., Chan K., Nørskov J. K., Cui Y., Wang H. (2017). Transition-Metal Single Atoms in a Graphene Shell as Active Centers for Highly Efficient Artificial Photosynthesis. Chem.

[cit5] Wang Y., Mao J., Meng X., Yu L., Deng D., Bao X. (2019). Catalysis with Two-Dimensional Materials Confining Single Atoms: Concept, Design, and Applications. Chem. Rev..

[cit6] Zhang H., Hwang S., Wang M., Feng Z., Karakalos S., Luo L., Qiao Z., Xie X., Wang C., Su D., Shao Y., Wu G. (2017). Single Atomic Iron Catalysts for Oxygen Reduction in Acidic Media: Particle Size Control and Thermal Activation. J. Am. Chem. Soc..

[cit7] Sun T., Xu L., Wang D., Li Y. (2019). Metal organic frameworks derived single atom catalysts for electrocatalytic energy conversion. Nano Res..

[cit8] Xue Y., Huang B., Yi Y., Guo Y., Zuo Z., Li Y., Jia Z., Liu H., Li Y. (2018). Anchoring zero valence single atoms of nickel and iron on graphdiyne for hydrogen evolution. Nat. Commun..

[cit9] Cheng Y., Yang S., Jiang S. P., Wang S. (2019). Supported Single Atoms as New Class of Catalysts for Electrochemical Reduction of Carbon Dioxide. Small Methods.

[cit10] Wang T., Zhao Q., Fu Y., Lei C., Yang B., Li Z., Lei L., Wu G., Hou Y. (2019). Carbon-Rich Nonprecious Metal Single Atom Electrocatalysts for CO 2 Reduction and Hydrogen Evolution. Small Methods.

[cit11] Zhao D., Zhuang Z., Li Y. (2020). Atomic site electrocatalysts for water splitting, oxygen reduction and selective oxidation. Chem. Soc. Rev..

[cit12] Liu H., Peng X., Liu X. (2018). Single-Atom Catalysts for the Hydrogen Evolution Reaction. ChemElectroChem.

[cit13] Yang L., Cheng D., Xu H., Zeng X., Wan X., Shui J., Xiang Z., Cao D. (2018). Unveiling the high-activity origin of single-atom iron catalysts for oxygen reduction reaction. Proc. Natl. Acad. Sci. U. S. A..

[cit14] Jiang J., Sun F., Zhou S., Hu W., Zhang H., Dong J., Jiang Z., Zhao J., Li J., Yan W., Wang M. (2018). Atomic-level insight into super-efficient electrocatalytic oxygen evolution on iron and vanadium co-doped nickel (oxy)hydroxide. Nat. Commun..

[cit15] Gao C., Chen S., Wang Y., Wang J., Zheng X., Zhu J., Song L., Zhang W., Xiong Y. (2018). Heterogeneous Single-Atom Catalyst for Visible-Light-Driven High-Turnover CO2 Reduction: The Role of Electron Transfer. Adv. Mater..

[cit16] Chen P., Zhou T., Xing L., Xu K., Tong Y., Xie H., Zhang L., Yan W., Chu W., Wu C., Xie Y. (2017). Atomically Dispersed Iron-Nitrogen Species as Electrocatalysts for Bifunctional Oxygen Evolution and Reduction Reactions. Angew. Chem. Int. Ed..

[cit17] Zhao L., Zhang Y., Huang L. B., Liu X. Z., Zhang Q. H., He C., Wu Z. Y., Zhang L. J., Wu J., Yang W., Gu L., Hu J. S., Wan L. J. (2019). Cascade anchoring strategy for general mass production of high-loading single-atomic metal-nitrogen catalysts. Nat. Commun..

[cit18] Han J., Meng X., Lu L., Bian J., Li Z., Sun C. (2019). Single-Atom Fe-Nx-C as an Efficient Electrocatalyst for Zinc–Air Batteries. Adv. Funct. Mater..

[cit19] Lü F., Zhao S., Guo R., He J., Peng X., Bao H., Fu J., Han L., Qi G., Luo J., Tang X., Liu X. (2019). Nitrogen-coordinated single Fe sites for efficient electrocatalytic N2 fixation in neutral media. Nano Energy.

[cit20] Zhang C., Yang S., Wu J., Liu M., Yazdi S., Ren M., Sha J., Zhong J., Nie K., Jalilov A. S., Li Z., Li H., Yakobson B. I., Wu Q., Ringe E., Xu H., Ajayan P. M., Tour J. M. (2018). Electrochemical CO2 Reduction with Atomic Iron-Dispersed on Nitrogen-Doped Graphene. Adv. Energy Mater..

[cit21] Yang Z., Wang Y., Zhu M., Li Z., Chen W., Wei W., Yuan T., Qu Y., Xu Q., Zhao C., Wang X., Li P., Li Y., Wu Y., Li Y. (2019). Boosting Oxygen Reduction Catalysis with Fe–N4 Sites Decorated Porous Carbons toward Fuel Cells. ACS Catal..

[cit22] Jiang R., Li L., Sheng T., Hu G., Chen Y., Wang L. (2018). Edge-Site Engineering of Atomically Dispersed Fe-N4 by Selective C-N Bond Cleavage for Enhanced Oxygen Reduction Reaction Activities. J. Am. Chem. Soc..

[cit23] Serov A., Artyushkova K., Atanassov P. (2014). Fe-N-C Oxygen Reduction Fuel Cell Catalyst Derived from Carbendazim: Synthesis, Structure, and Reactivity. Adv. Energy Mater..

[cit24] Pan Y., Chen Y., Wu K., Chen Z., Liu S., Cao X., Cheong W. C., Meng T., Luo J., Zheng L., Liu C., Wang D., Peng Q., Li J., Chen C. (2019). Regulating the coordination structure of single-atom Fe-NxCy catalytic sites for benzene oxidation. Nat. Commun..

[cit25] Li Z., Zhuang Z., Lv F., Zhu H., Zhou L., Luo M., Zhu J., Lang Z., Feng S., Chen W., Mai L., Guo S. (2018). The Marriage of the FeN4 Moiety and MXene Boosts Oxygen Reduction Catalysis: Fe 3d Electron Delocalization Matters. Adv. Mater..

[cit26] Zhu Y., Zhang B., Liu X., Wang D. W., Su D. S. (2014). Unravelling the structure of electrocatalytically active Fe-N complexes in carbon for the oxygen reduction reaction. Angew. Chem. Int. Ed..

[cit27] Zheng Y., Jiao Y., Zhu Y., Cai Q., Vasileff A., Li L. H., Han Y., Chen Y., Qiao S. Z. (2017). Molecule-Level g-C3N4 Coordinated Transition Metals as a New Class of Electrocatalysts for Oxygen Electrode Reactions. J. Am. Chem. Soc..

[cit28] Asnavandi M., Yin Y., Li Y., Sun C., Zhao C. (2018). Promoting Oxygen Evolution Reactions through Introduction of Oxygen Vacancies to Benchmark NiFe–OOH Catalysts. ACS Energy Lett..

[cit29] Li Y. F., Selloni A. (2014). Mechanism and Activity of Water Oxidation on Selected Surfaces of Pure and Fe-Doped NiOx. ACS Catal..

[cit30] Sa Y. J., Seo D. J., Woo J., Lim J. T., Cheon J. Y., Yang S. Y., Lee J. M., Kang D., Shin T. J., Shin H. S., Jeong H. Y., Kim C. S., Kim M. G., Kim T. Y., Joo S. H. (2016). A General Approach to Preferential Formation of Active Fe-Nx Sites in Fe-N/C Electrocatalysts for Efficient Oxygen Reduction Reaction. J. Am. Chem. Soc..

[cit31] Zhu Q.-L., Xia W., Zheng L.-R., Zou R., Liu Z., Xu Q. (2017). Atomically Dispersed Fe/N-Doped Hierarchical Carbon Architectures Derived from a Metal–Organic Framework Composite for Extremely Efficient Electrocatalysis. ACS Energy Lett..

[cit32] Zhang H., Chung H. T., Cullen D. A., Wagner S., Kramm U. I., More K. L., Zelenay P., Wu G. (2019). High-performance fuel cell cathodes exclusively containing atomically dispersed iron active sites. Energy Environ. Sci..

[cit33] Xiao M., Zhu J., Ma L., Jin Z., Ge J., Deng X., Hou Y., He Q., Li J., Jia Q., Mukerjee S., Yang R., Jiang Z., Su D., Liu C., Xing W. (2018). Microporous Framework Induced Synthesis of Single-Atom Dispersed Fe-N-C Acidic ORR Catalyst and Its in Situ Reduced Fe-N4 Active Site Identification Revealed by X-ray Absorption Spectroscopy. ACS Catal..

[cit34] Gu J., Hsu C. S., Bai L., Chen H. M., Hu X. (2019). Atomically dispersed Fe(3+) sites catalyze efficient CO2 electroreduction to CO. Science.

[cit35] Zhang R., Jiao L., Yang W. J., Wan G., Jiang H. L. (2019). Single-atom catalysts templated by metal–organic frameworks for electrochemical nitrogen reduction. J. Mater. Chem. A.

[cit36] Gu W. L., Wang H. J., Jiao L., Wu Y., Chen Y. X., Hu L. Y., Gong J. M., Du D., Zhu C. Z. (2020). Single-Atom Iron Boosts Electrochemiluminescence. Angew. Chem. Int. Ed..

[cit37] Li Z., Sun H., Wei L., Jiang W. J., Wu M., Hu J. S. (2017). Lamellar Metal Organic Framework-Derived Fe-N-C Non-Noble Electrocatalysts with Bimodal Porosity for Efficient Oxygen Reduction. ACS Appl. Mater. Interfaces.

[cit38] Fu S., Zhu C., Su D., Song J., Yao S., Feng S., Engelhard M. H., Du D., Lin Y. (2018). Porous Carbon-Hosted Atomically Dispersed Iron-Nitrogen Moiety as Enhanced Electrocatalysts for Oxygen Reduction Reaction in a Wide Range of pH. Small.

[cit39] Zhu C., Fu S., Song J., Shi Q., Su D., Engelhard M. H., Li X., Xiao D., Li D., Estevez L., Du D., Lin Y. (2017). Self-Assembled Fe-N-Doped Carbon Nanotube Aerogels with Single-Atom Catalyst Feature as High-Efficiency Oxygen Reduction Electrocatalysts. Small.

[cit40] Mun Y., Lee S., Kim K., Kim S., Lee S., Han J. W., Lee J. (2019). Versatile Strategy for Tuning ORR Activity of a Single Fe-N4 Site by Controlling Electron-Withdrawing/Donating Properties of a Carbon Plane. J. Am. Chem. Soc..

[cit41] Sa Y. J., Seo D.-J., Woo J., Lim J. T., Cheon J. Y., Yang S. Y., Lee J. M., Kang D., Shin T. J., Shin H. S., Jeong H. Y., Kim C. S., Kim M. G., Kim T.-Y., Joo S. H. (2016). A General Approach to Preferential Formation of Active Fe–Nx Sites in Fe–N/C Electrocatalysts for Efficient Oxygen Reduction Reaction. J. Am. Chem. Soc..

[cit42] Varela A. S., Ranjbar Sahraie N., Steinberg J., Ju W., Oh H. S., Strasser P. (2015). Metal-Doped Nitrogenated Carbon as an Efficient Catalyst for Direct CO2 Electroreduction to CO and Hydrocarbons. Angew. Chem. Int. Ed..

[cit43] Choi C. H., Baldizzone C., Polymeros G., Pizzutilo E., Kasian O., Schuppert A. K., Ranjbar Sahraie N., Sougrati M.-T., Mayrhofer K. J. J., Jaouen F. (2016). Minimizing Operando Demetallation of Fe-N-C Electrocatalysts in Acidic Medium. ACS Catal..

[cit44] Jiang W. J., Gu L., Li L., Zhang Y., Zhang X., Zhang L. J., Wang J. Q., Hu J. S., Wei Z., Wan L. J. (2016). Understanding the High Activity of Fe-N-C Electrocatalysts in Oxygen Reduction: Fe/Fe3C Nanoparticles Boost the Activity of Fe-N(x). J. Am. Chem. Soc..

[cit45] Zhou B., Liu L., Cai P., Zeng G., Li X., Wen Z., Chen L. (2017). Ferrocene-based porous organic polymer derived high-performance electrocatalysts for oxygen reduction. J. Mater. Chem. A.

[cit46] Mei D. L., Yuan X. X., Ma Z., Wei P., Yu X. B., Yang J., Ma Z. F. (2016). A SnO2-Based Cathode Catalyst for Lithium-Air Batteries. ACS Appl. Mater. Interfaces.

[cit47] Yu Q. Y., Yin S., Zhang J., Yin H. M. (2019). Structure dependent activity and durability towards oxygen reduction reaction on Pt modified nanoporous gold. Electrochim. Acta.

[cit48] Shao M. H., Chang Q. W., Dodelet J. P., Chenitz R. (2016). Recent Advances in Electrocatalysts for Oxygen Reduction Reaction. Chem. Rev..

[cit49] Gomez J. C. C., Moliner R., Lazaro M. J. (2016). Palladium-Based Catalysts as Electrodes for Direct Methanol Fuel Cells: A Last Ten Years Review. Catalysts.

[cit50] Jin H. Y., Guo C. X., Liu X., Liu J. L., Vasileff A., Jiao Y., Zheng Y., Qiao S. Z. (2018). Emerging Two-Dimensional Nanomaterials for Electrocatalysis. Chem. Rev..

[cit51] Stacy J., Regmi Y. N., Leonard B., Fan M. H. (2017). The recent progress and future of oxygen reduction reaction catalysis: A review. Renewable Sustainable Energy Rev..

[cit52] Fabbri E., Habereder A., Waltar K., Kotz R., Schmidt T. J. (2014). Developments and perspectives of oxide-based catalysts for the oxygen evolution reaction. Catal. Sci. Technol..

[cit53] Jiao Y., Zheng Y., Jaroniec M. T., Qiao S. Z. (2015). Design of electrocatalysts for oxygen- and hydrogen-involving energy conversion reactions. Chem. Soc. Rev..

[cit54] Shen G., Zhang R., Pan L., Hou F., Zhao Y., Shen Z., Mi W., Shi C., Wang Q., Zhang X., Zou J. J. (2020). Regulating the Spin State of Fe(III) by Atomically Anchoring on Ultrathin Titanium Dioxide for Efficient Oxygen Evolution Electrocatalysis. Angew. Chem. Int. Ed..

[cit55] Kortlever R., Shen J., Schouten K. J. P., Calle-Vallejo F., Koper M. T. M. (2015). Catalysts and Reaction Pathways for the Electrochemical Reduction of Carbon Dioxide. J. Phys. Chem. Lett..

[cit56] Centi G., Perathoner S. (2009). Opportunities and prospects in the chemical recycling of carbon dioxide to fuels. Catal. Today.

[cit57] Hori Y., Wakebe H., Tsukamoto T., Koga O. (1994). Electrocatalytic Process of Co Selectivity in Electrochemical Reduction of Co2 at Metal-Electrodes in Aqueous-Media. Electrochim. Acta.

[cit58] Huan T. N., Ranjbar N., Rousse G., Sougrati M., Zitolo A., Mougel V., Jaouen F., Fontecave M. (2017). Electrochemical Reduction of CO2 Catalyzed by Fe-N-C Materials: A Structure–Selectivity Study. ACS Catal..

[cit59] Ju W., Bagger A., Hao G. P., Varela A. S., Sinev I., Bon V., Roldan Cuenya B., Kaskel S., Rossmeisl J., Strasser P. (2017). Understanding activity and selectivity of metal-nitrogen-doped carbon catalysts for electrochemical reduction of CO2. Nat. Commun..

[cit60] Ren X., Zhao J. X., Wei Q., Ma Y. J., Guo H. R., Liu Q., Wang Y., Cui G. W., Asiri A. M., Li B. H., Tang B., Sun X. P. (2019). High-Performance N-2-to-NH3 Conversion Electrocatalyzed by Mo2C Nanorod. ACS Cent. Sci..

[cit61] Shipman M. A., Symes M. D. (2017). Recent progress towards the electrosynthesis of ammonia from sustainable resources. Catal. Today.

[cit62] Skulason E., Bligaard T., Gudmundsdottir S., Studt F., Rossmeisl J., Abild-Pedersen F., Vegge T., Jonsson H., Norskov J. K. (2012). A theoretical evaluation of possible transition metal electro-catalysts for N-2 reduction. Phys. Chem. Chem. Phys..

[cit63] Zhang R., Jiao L., Yang W., Wan G., Jiang H.-L. (2019). Single-atom catalysts templated by metal–organic frameworks for electrochemical nitrogen reduction. J. Mater. Chem. A.

[cit64] Wei Y. S., Zhang M., Zou R., Xu Q. (2020). Metal-Organic Framework-Based Catalysts with Single Metal Sites. Chem. Rev..

[cit65] Shen H. J., Gracia-Espino E., Ma J. Y., Zang K. T., Luo J., Wang L., Gao S. S., Mamat X., Hu G. Z., Wagberg T., Guo S. J. (2017). Synergistic Effects between Atomically Dispersed Fe-N-C and C-S-C for the Oxygen Reduction Reaction in Acidic Media. Angew. Chem. Int. Ed..

[cit66] Song P., Wang Y., Pan J., Xu W. L., Zhuang L. (2015). Structure-activity relationship in high-performance iron-based electrocatalysts for oxygen reduction reaction. J. Power Sources.

